# PhagePhisher: a pipeline for the discovery of covert viral sequences in complex genomic datasets

**DOI:** 10.1099/mgen.0.000053

**Published:** 2016-03-10

**Authors:** Thomas Hatzopoulos, Siobhan C. Watkins, Catherine Putonti

**Affiliations:** Loyola University Chicago, Chicago, IL, USA

**Keywords:** bacteriophage, metagenomics, metaviromics, prophage, whole genome sequencing, virus

## Abstract

Obtaining meaningful viral information from large sequencing datasets presents unique challenges distinct from prokaryotic and eukaryotic sequencing efforts. The difficulties surrounding this issue can be ascribed in part to the genomic plasticity of viruses themselves as well as the scarcity of existing information in genomic databases. The open-source software PhagePhisher (http://www.putonti-lab.com/phagephisher) has been designed as a simple pipeline to extract relevant information from complex and mixed datasets, and will improve the examination of bacteriophages, viruses, and virally related sequences, in a range of environments. Key aspects of the software include speed and ease of use; PhagePhisher can be used with limited operator knowledge of bioinformatics on a standard workstation. As a proof-of-concept, PhagePhisher was successfully implemented with bacteria–virus mixed samples of varying complexity. Furthermore, viral signals within microbial metagenomic datasets were easily and quickly identified by PhagePhisher, including those from prophages as well as lysogenic phages, an important and often neglected aspect of examining phage populations in the environment. PhagePhisher resolves viral-related sequences which may be obscured by or imbedded in bacterial genomes.

## Data Summary

Raw sequencing data examined here have either been deposited in GenBank (phage ϕVader) under accession number KT254130 (http://www.ncbi.nlm.nih.gov/nuccore/KT254130) (Data Citation 1) or are publicly available through our website: http://www.putonti-lab.com/phagephisher. Source code is publicly available through our website: http://www.putonti-lab.com/phagephisher, including a sample fastq file for analysis. Genomic sequences and annotations for analyses were retrieved from the NCBI RefSeq data collection (Data Citation 2) and are listed in File S1 (available in the online Supplementary Material).

## Impact Statement

Despite their abundance and ubiquity, genomic data from environmental viruses are relatively scant in the public repositories. Cultivating and/or producing pure viral isolates in the lab presents practical difficulties, and even with current and forthcoming high-throughput sequencing technologies, it is challenging to identify sequences that are truly viral in origin. The PhagePhisher pipeline presented here provides a computational solution for efficiently and effectively identifying viral sequences. Key aspects of PhagePhisher are expediency and flexibility; analyses can be performed locally and necessitate minimal computational expertise. This technique will thus allow those studying viruses to better examine genomes which are inherently prone to high incidence of host signal contamination, whether it originates in culture or at the genomic level. While our proof-of-concept work analyses three difficult datasets containing phages, PhagePhisher can also be applied to eukaryotic-infecting viruses. As such, some of the difficulties hindering environmental viromic investigations are eliminated.

## Introduction

Bacteriophages, or phages, are the most abundant entities on Earth, preying on bacteria in a range of niches across the planet (e.g. Mizuno *et al.*, 2013; Yoshida *et al.*, 2013) as well as within the ecology of the human microbiome (e.g. Abeles & Pride, 2014; Dutilh *et al.*, 2014). Given the multitude of niches they inhabit, it is not surprising that phages are extremely diverse. Phages mediate mortality and drive bacterial diversity (Clokie *et al.*, 2011; Jacquet *et al.*, 2010) and therefore have incredible potential to impact bacterial metabolism and processes such as nutrient cycling on a global scale (Bratbak *et al.*, 1994; Wilhelm & Suttle, 1999). Metaviromic datasets (e.g. Bolduc *et al.*, 2012; Hurwitz & Sullivan, 2013; Reyes *et al.*, 2015; Rodriguez-Valera *et al.*, 2014; Santos *et al.*, 2010) have uncovered countless putative novel phage genes which exhibit no similarity to existing sequences in databases, the function of which can only be speculated at this stage in our understanding. Nevertheless, there exists a considerable paucity of information for phages on a genomic and phenotypic scale (Rohwer & Edwards, 2002). Part of the reason such a disparity exists parallels issues regarding culture-based environmental bacteriology studies.

Environmental viral genomics is, however, not without its challenges; isolating reads that are viral in origin from host-associated nucleic acid is problematic. Some phages are very difficult to sequence, e.g. *Pseudomonas aeruginosa* phage PaP1 took over 10 years to complete (Lu *et al.*, 2014). Furthermore, considerable problems may arise during assembly and annotation as a result of the inherent genetic mosaicism of phages (Born *et al.*, 2011); in fact, genome mosaicism appears to be a general feature of all viruses, not just phages (Jachiet *et al.*, 2014). To date, phage viromic studies have been heavily biased towards the examination of lytic phages; therefore, only gathering a glimpse into the rich diversity of phages. Metagenomic whole genome sequencing (WGS) surveys of microbial communities, nevertheless, do capture some of these viral signals. However, viral nucleic acids are generally outnumbered by those of bacterial in origin; furthermore, discerning between prophage sequences embedded within the prokaryotic genome and autonomous viral sequences is far from trivial. While a number of prophage detection tools are available (e.g. Akhter *et al.*, 2012; Lima-Mendez *et al.*, 2008; Zhou *et al.*, 2011), identifying viral sequences within heterogeneous samples is more problematic; one solution, VirSorter (Roux *et al.*, 2015), detects putative prophage as well as viral sequences given their homology and/or virus-like structure relative to available viral sequence data.

PhagePhisher pipeline has been designed as a method to extract obscure viral-specific sequences from data produced by WGS and reassemble it to more closely describe the virus(es) of interest. In contrast to VirSorter (Roux *et al.*, 2015), PhagePhisher tackles the task at hand by leveraging existing knowledge about what is *not* viral. Taking a subtractive approach akin to decontamination tools, e.g. DeconSeq (Schmieder & Edwards, 2011), PhagePhisher can extract and assemble any viral sequence(s) of interest, be it from high-throughput sequencing of single isolates, complex viral communities, or mixed (bacterial and viral) microbial communities including prophages and lysogenic species. Three proof-of-concept studies were performed representative of various ‘signal-to-noise’ (viral to non-viral DNA) scenarios for environmental phage datasets, exemplifying the effectiveness and versatility of the PhagePhisher pipeline.

## Theory and Implementation

**Fig. 1.**
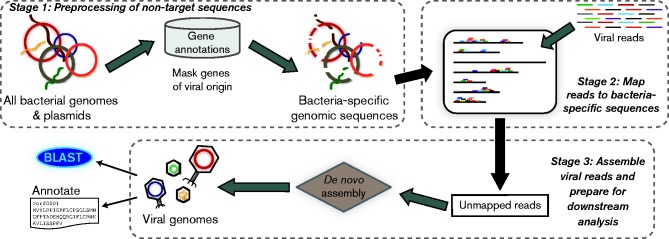
Schematic of the PhagePhisher pipeline.

The PhagePhisher pipeline, written in Python, is a three-step process which integrates new functionality as well as repurposes existing software to isolate viral sequences from high-throughput sequencing datasets (Fig. 1). Firstly, all non-target, e.g. contaminant and/or host species, genomic sequences are collected and processed. The user can select which sequence(s) to use as well as select to mask sequences of viral origin within this genome(s), e.g. prophages, as illustrated in Fig. 1. (Coding regions annotated as ‘phage’ or ‘viral’ in origin are replaced with Ns; see the PhagePhisher's ReadMe for further details.) The collection of non-target-specific sequence(s) is henceforth referred to as the ‘background genome’. Next, viral WGS reads are mapped to the background; the parameters for mapping, however, are set to be tolerant of mismatches accommodating variations between sequenced strains and bacterial isolates in nature. Lastly, those reads which do not resemble the non-target collection – the unmapped reads – are assembled and ready for downstream analysis such as evaluation via blast and/or annotation. While here Stages 2 and 3 are performed using the tools Bowtie2 (Langmead & Salzberg, 2012) and Velvet (Zerbino & Birney, 2008), respectively, the plug-and-play nature of PhagePhisher can easily accommodate any mapping and *de novo* assembly strategy. Bowtie2 (Langmead & Salzberg, 2012) in Stage 2 facilities PhagePhisher's consideration of large background genomes with a small memory footprint.

The following three proof-of-concept studies highlight the agility of the pipeline. Full details regarding the protocols for the following examples can be found in File S2 (available in the online Supplementary Material).

### Case Study 1: separating viral reads from host genomic sequences

Whole genome sequencing of an environmental *Pseudomonas* sp. phage (ϕVader) was conducted (Malki *et al.*, 2015b). Neither the phage nor the laboratory host *P. aeruginosa* ATCC 15692 have complete genomic sequences available. As such, the RefSeq *P. aeruginosa* PAO1 genome (GenBank: NC_002516), excluding annotated bacteriophage coding regions, was used to separate host-derived sequences from those from the phage genome. Of the 2.9 million paired-end reads generated, 69 % mapped (either one or both of the paired-end reads) to the *P. aeruginosa* genome. The remaining 97 614 paired-end reads were then assembled (Table 1).

**Table 1:** Statistics for the performance of the PhagePhisher Pipeline

Case study	No. of bp	Percentage of bp predicted to belong to background	Final contigs assembled ( ≥ 1000 bp)			
			No.	Max. length	N50	No. of bp
1. *Pseudomonas* phage and host	1.5 Gbp	69.30 %	70	66 379	1654	148 540
2. ϕMHI42, host and contaminants	42 Mbp	38.42 %	4851	17 225	2928	12 228 615
3. Lysogenic phages in bacterial WGS	66 Mbp	71.13 %	2	5400	5400	6487

This compact study confirmed that PhagePhisher could be used satisfactorily to separate viral and bacterial genomic data. In the event in which the host genome is sequenced and assembled (or at minimum a near-neighbour as was used here), essentially all reads belonging to the host species would be removed. Inclusion of additional *Pseudomonas* genomes, rather than just the one considered here, would likely reduce the number of reads passing through Stage 2. Nevertheless, the complete genome of ϕVader was able to be assembled; its genome (GenBank: KT254130) is within the longest contig assembled for this dataset (Table 1). All other contigs are host sequences (as determined through the BLASTing of the individual contigs). (Note, ϕVader's genome is slightly smaller than the assembled contig due to terminal redundancy.)

### Case Study 2: isolating viral reads from unknown contaminating DNAs

For certain types of phage, contaminating bacterial DNA may originate in organisms other than the host, e.g. those bacterial hosts which are difficult to maintain in axenic culture. This presents a far more complex task with regard to the isolation of viral genomic information. Previously, the authors have experienced some difficulty in this with the freshwater cyanophage ϕMHI42 (Watkins *et al.*, 2014). While ϕMHI42 has been characterized, the genomic sequence remains unknown. The background genome created included all publicly available bacterial genomes and plasmids less those from the phylum *Cyanobacteria* as many cyanophages are known to possess auxiliary genes similar to those found in cyanobacteria (Millard *et al.*, 2004); thus, discerning between phage and host sequences is problematic. DNA extracted from phage lysate (negative for 16S rRNA gene amplification, indicative of minimal levels of bacterial genomic material) was sequenced via the 454 platform. Again, phage-related coding regions were masked out and reads generated from the sequencing of ϕMHI42 were screened and assembled (Table 1).

In contrast to the analysis of ϕVader, the complete genome is not obtained within a single contig. The contigs were further analysed using the rast web service (Overbeek *et al.*, 2014) for predicting ORFs and their putative functionality; blastx searches for the predicted ORFs were also conducted. Using the PhagePhisher pipeline, it was possible to identify annotations of interest, allowing for an estimation of some aspects of the genomic nature of ϕMHI42. Of particular interest was the presence of photolyases (enzymes which repair DNA after damage by exposure to UV light), genes relating to toxin production, phage structural proteins, phage antirepressors and phage recombinases: highly interesting considering ϕMHI42 is a broad-host-range phage (Watkins *et al.*, 2014).

ϕMHI42 is a large phage, with a genome size previously estimated at 150 kbp via pulsed field gel electrophoresis (PFGE) – far larger than any single contig produced here (Table 1). It is particularly difficult to manipulate and examine in the laboratory; extraction and purification of sufficient quantities of DNA from virally induced lysate was extremely difficult before factoring in the presence of such a high quantity of contaminating bacterial DNA. These issues combined meant it was not possible to reconstruct the entire genome for this phage. However, a great deal more insight was obtained as a result of using PhagePhisher than had been seen previously. For a smaller phage, which is easier to handle in the laboratory, the PhagePhisher pipeline could be used to reconstruct an entire genome in the same fashion.

### Case Study 3: identifying lysogenic phages from bacterial populations

Metagenomic surveys of bacteriophage populations in nature are predominantly limited to those operating within the lytic cycle. Identifying virus-like particles within bacterial WGS studies is typically dependent upon blast searches. The PhagePhisher pipeline can quickly assist in separating the two. The raw sequencing paired-end reads generated from a WGS survey of the nearshore waters of Lake Michigan were screened against all publicly available bacterial genomes and plasmids (again with all annotated viral/phage sequences removed from consideration). (Bacterial cells were isolated via size, 0.22 μl filtration; for details see Malki *et al.*, 2015a.) In contrast to our first case study, here paired-end reads were considered individually; thus individual reads which mapped to the background were removed and unmapped reads were considered singletons and subsequently assembled (Table 1). Given the complexity of the environment and the shallow sequencing performed for this proof-of-concept, it is not surprising that the N50 value was only slightly better than the read size itself (150 bp).

To assess the ability of the PhagePhisher tool to identify lysogenic phages isolated from bacterial cells, the five largest contigs were selected and BLASTed against the nr/nt database. The search was not limited to any particular organism or taxa. All five of these contigs produced statistically significant hits (as assessed via the E-value) to phage sequences, including: Bacteriophage S13 (GenBank: M14428) (E-value = 0), Cyanophage Syn2 (GenBank: HQ634190]) (E-value = 6e − 10), and Enterococcus phage VD13 (GenBank: KJ094032) (E-value = 4e − 10); in fact, two of the contigs showed homology to the Enterococcus phage VD13. Moreover, the blast search for one of the contigs revealed no significant similarity to the database. By referencing the annotation of the cyanophage genome, the contig was found to be homologous (even more so at the amino acid level) to the phage's annotated recombination endonuclease. The fact that the contigs did not find homology with bacterial sequences further validates the use of the tool to isolate viral sequences.

## Conclusion

PhagePhisher was successfully used as a pipeline to analyse three types of datasets, sequences obtained from: a clonal population of a *Pseudomonas* phage sequenced in combination with a small quantity of host DNA; a sequenced sample containing a heavily bacterially contaminated, hard-to-sequence cyanophage that was resolved to a degree not previously possible; and a microbial metagenomic dataset containing a variety of lytic and lysogenic phages. PhagePhisher may be used to construct whole phage genomes from mixed information, which will be particularly useful in the examination of ‘hard-to-sequence’ phages where enough coverage is obtained. PhagePhisher is an intuitive pipeline which may be used with a small previous knowledge of bioinformatics, improving its accessibility to biologists.

The PhagePhisher pipeline can easily be adapted as new bioinformatic analysis tools become available. Furthermore, additional downstream tools, e.g. scaffolding software (e.g. Boetzer *et al.*, 2011), can assist in the finishing of the genome sequence(s) produced. The pipeline presented here is expedient; run-time from beginning to end is just a little over an hour. Most importantly, person-hours are saved, as researchers must only inspect a handful of sequences in comparison with those from the full run. While applied here to phage sequence analysis, the same methodology can be applied to any sequencing project, be it viral, bacterial or protistan in origin.

## Supplementary Data

433 Supplementary Data
