# Supplementary material for: PhagePhisher: a pipeline for the discovery of covert viral sequences in complex genomic datasets
Source: Microb Genom. 2016 Mar 10;2(3):e000053. doi: 10.1099/mgen.0.000053 (PMC5320576; doi:10.1099/mgen.0.000053)
Supplement: Supplementary file 1 — Supplementary Data [file mgen-02-53-s001.pdf]

SUPPLEMENTARY FILE 1: GENOME SEQUENCES USED FOR ANALYSES

GenBank Accession Numbers. Sequences and annotations for all of the listed accession numbers were used in the background genome for Case Study 3 (Identifying Lysogenic Phages from Bacterial Populations). This same set was used for Case Study 2 (Isolating Viral Reads from Unknown Contaminating DNAs) with the exception of those indicated in green.

NC_000117	NC_008265	NC_010721	NC_013739	NC_015707	NC_017333	NC_018633	NC_021151
NC_000853	NC_008268	NC_010723	NC_013740	NC_015709	NC_017334	NC_018634	NC_021155
NC_000854	NC_008269	NC_010724	NC_013741	NC_015710	NC_017335	NC_018635	NC_021156
NC_000868	NC_008270	NC_010725	NC_013742	NC_015711	NC_017336	NC_018636	NC_021157
NC_000907	NC_008271	NC_010727	NC_013743	NC_015713	NC_017337	NC_018637	NC_021169
NC_000908	NC_008273	NC_010729	NC_013744	NC_015714	NC_017338	NC_018638	NC_021171
NC_000909	NC_008274	NC_010730	NC_013745	NC_015715	NC_017339	NC_018639	NC_021172
NC_000911	NC_008277	NC_010740	NC_013746	NC_015716	NC_017340	NC_018640	NC_021173
NC_000912	NC_008278	NC_010741	NC_013747	NC_015717	NC_017341	NC_018641	NC_021174
NC_000913	NC_008309	NC_010742	NC_013748	NC_015722	NC_017342	NC_018642	NC_021175
NC_000914	NC_008312	NC_010793	NC_013749	NC_015723	NC_017343	NC_018643	NC_021176
NC_000915	NC_008313	NC_010794	NC_013757	NC_015724	NC_017344	NC_018644	NC_021177
NC_000916	NC_008314	NC_010801	NC_013766	NC_015725	NC_017345	NC_018645	NC_021179
NC_000917	NC_008319	NC_010802	NC_013767	NC_015726	NC_017346	NC_018646	NC_021181
NC_000918	NC_008320	NC_010803	NC_013768	NC_015727	NC_017347	NC_018649	NC_021182
NC_000919	NC_008321	NC_010804	NC_013769	NC_015728	NC_017348	NC_018650	NC_021183
NC_000921	NC_008322	NC_010805	NC_013770	NC_015729	NC_017349	NC_018651	NC_021184
NC_000922	NC_008340	NC_010813	NC_013771	NC_015730	NC_017350	NC_018652	NC_021191
NC_000948	NC_008341	NC_010814	NC_013790	NC_015731	NC_017351	NC_018654	NC_021192
NC_000949	NC_008342	NC_010815	NC_013791	NC_015732	NC_017352	NC_018655	NC_021193
NC_000950	NC_008343	NC_010816	NC_013792	NC_015733	NC_017353	NC_018656	NC_021194
NC_000951	NC_008344	NC_010830	NC_013793	NC_015734	NC_017354	NC_018657	NC_021195
NC_000952	NC_008345	NC_010831	NC_013798	NC_015735	NC_017355	NC_018658	NC_021200
NC_000953	NC_008346	NC_010842	NC_013799	NC_015736	NC_017356	NC_018659	NC_021213
NC_000954	NC_008347	NC_010843	NC_013849	NC_015737	NC_017357	NC_018660	NC_021214
NC_000955	NC_008358	NC_010844	NC_013850	NC_015738	NC_017358	NC_018661	NC_021215
NC_000956	NC_008369	NC_010845	NC_013851	NC_015740	NC_017359	NC_018662	NC_021216
NC_000957	NC_008378	NC_010846	NC_013852	NC_015741	NC_017360	NC_018663	NC_021217
NC_000958	NC_008379	NC_010935	NC_013853	NC_015744	NC_017361	NC_018664	NC_021218
NC_000959	NC_008380	NC_010939	NC_013854	NC_015756	NC_017362	NC_018665	NC_021219
NC_000961	NC_008381	NC_010940	NC_013855	NC_015757	NC_017363	NC_018666	NC_021224
NC_000962	NC_008382	NC_010941	NC_013856	NC_015758	NC_017364	NC_018672	NC_021225
NC_000963	NC_008383	NC_010942	NC_013857	NC_015759	NC_017365	NC_018673	NC_021226
NC_000964	NC_008384	NC_010943	NC_013858	NC_015760	NC_017366	NC_018674	NC_021227
NC_001263	NC_008385	NC_010981	NC_013859	NC_015761	NC_017367	NC_018675	NC_021228
NC_001264	NC_008386	NC_010994	NC_013860	NC_015844	NC_017368	NC_018676	NC_021231
NC_001318	NC_008387	NC_010995	NC_013861	NC_015845	NC_017369	NC_018677	NC_021232
NC_001399	NC_008388	NC_010996	NC_013862	NC_015846	NC_017370	NC_018678	NC_021233
NC_001732	NC_008389	NC_010997	NC_013887	NC_015847	NC_017371	NC_018679	NC_021234
NC_001733	NC_008390	NC_010998	NC_013888	NC_015848	NC_017372	NC_018680	NC_021235
NC_001773	NC_008391	NC_010999	NC_013889	NC_015850	NC_017373	NC_018681	NC_021236
NC_001849	NC_008392	NC_011000	NC_013890	NC_015851	NC_017374	NC_018682	NC_021237
NC_001850	NC_008435	NC_011001	NC_013891	NC_015852	NC_017375	NC_018683	NC_021251
NC_001851	NC_008463	NC_011002	NC_013892	NC_015853	NC_017376	NC_018684	NC_021252
NC_001852	NC_008496	NC_011003	NC_013893	NC_015854	NC_017377	NC_018685	NC_021277
NC_001853	NC_008497	NC_011004	NC_013894	NC_015856	NC_017378	NC_018686	NC_021278
NC_001854	NC_008498	NC_011025	NC_013895	NC_015857	NC_017379	NC_018687	NC_021279
NC_001855	NC_008499	NC_011026	NC_013921	NC_015858	NC_017380	NC_018688	NC_021280

NC_001856	NC_008500	NC_011027	NC_013922	NC_015859	NC_017381	NC_018689	NC_021281
NC_001857	NC_008501	NC_011034	NC_013923	NC_015865	NC_017382	NC_018690	NC_021282
NC_001869	NC_008502	NC_011035	NC_013924	NC_015866	NC_017383	NC_018691	NC_021283
NC_001880	NC_008503	NC_011047	NC_013925	NC_015873	NC_017384	NC_018692	NC_021284
NC_001903	NC_008504	NC_011059	NC_013926	NC_015875	NC_017385	NC_018693	NC_021285
NC_001904	NC_008505	NC_011060	NC_013928	NC_015876	NC_017386	NC_018694	NC_021286
NC_001988	NC_008506	NC_011061	NC_013929	NC_015903	NC_017387	NC_018695	NC_021287
NC_002127	NC_008507	NC_011071	NC_013930	NC_015904	NC_017388	NC_018696	NC_021288
NC_002128	NC_008508	NC_011072	NC_013939	NC_015905	NC_017389	NC_018697	NC_021289
NC_002162	NC_008509	NC_011079	NC_013940	NC_015906	NC_017390	NC_018698	NC_021290
NC_002163	NC_008510	NC_011080	NC_013941	NC_015907	NC_017391	NC_018699	NC_021291
NC_002179	NC_008511	NC_011081	NC_013942	NC_015908	NC_017392	NC_018700	NC_021294
NC_002180	NC_008512	NC_011082	NC_013943	NC_015909	NC_017393	NC_018701	NC_021295
NC_002182	NC_008513	NC_011083	NC_013946	NC_015910	NC_017394	NC_018704	NC_021313
NC_002252	NC_008525	NC_011092	NC_013947	NC_015911	NC_017395	NC_018706	NC_021314
NC_002253	NC_008526	NC_011093	NC_013948	NC_015913	NC_017396	NC_018707	NC_021350
NC_002488	NC_008527	NC_011094	NC_013949	NC_015914	NC_017397	NC_018708	NC_021351
NC_002489	NC_008528	NC_011126	NC_013954	NC_015915	NC_017398	NC_018709	NC_021352
NC_002490	NC_008529	NC_011134	NC_013956	NC_015916	NC_017399	NC_018712	NC_021353
NC_002491	NC_008530	NC_011138	NC_013957	NC_015917	NC_017400	NC_018719	NC_021354
NC_002505	NC_008531	NC_011143	NC_013958	NC_015918	NC_017401	NC_018720	NC_021355
NC_002506	NC_008532	NC_011144	NC_013959	NC_015919	NC_017402	NC_018721	NC_021361
NC_002516	NC_008533	NC_011145	NC_013960	NC_015920	NC_017403	NC_018722	NC_021362
NC_002528	NC_008536	NC_011146	NC_013961	NC_015921	NC_017404	NC_018742	NC_021485
NC_002570	NC_008537	NC_011147	NC_013962	NC_015922	NC_017405	NC_018743	NC_021486
NC_002578	NC_008538	NC_011148	NC_013964	NC_015930	NC_017406	NC_018744	NC_021487
NC_002607	NC_008539	NC_011149	NC_013965	NC_015931	NC_017407	NC_018745	NC_021490
NC_002608	NC_008541	NC_011184	NC_013966	NC_015942	NC_017408	NC_018746	NC_021491
NC_002620	NC_008542	NC_011185	NC_013967	NC_015943	NC_017409	NC_018747	NC_021492
NC_002655	NC_008543	NC_011186	NC_013968	NC_015944	NC_017410	NC_018748	NC_021494
NC_002662	NC_008544	NC_011204	NC_013971	NC_015945	NC_017411	NC_018749	NC_021495
NC_002663	NC_008545	NC_011205	NC_013972	NC_015946	NC_017412	NC_018750	NC_021496
NC_002677	NC_008553	NC_011206	NC_013973	NC_015947	NC_017413	NC_018751	NC_021497
NC_002678	NC_008554	NC_011224	NC_013974	NC_015948	NC_017414	NC_018828	NC_021498
NC_002679	NC_008555	NC_011226	NC_014000	NC_015949	NC_017415	NC_018829	NC_021499
NC_002682	NC_008563	NC_011229	NC_014002	NC_015951	NC_017416	NC_018830	NC_021500
NC_002689	NC_008564	NC_011244	NC_014004	NC_015952	NC_017417	NC_018866	NC_021501
NC_002695	NC_008565	NC_011245	NC_014005	NC_015953	NC_017418	NC_018867	NC_021503
NC_002696	NC_008566	NC_011246	NC_014006	NC_015954	NC_017419	NC_018868	NC_021504
NC_002737	NC_008567	NC_011247	NC_014007	NC_015955	NC_017420	NC_018870	NC_021505
NC_002745	NC_008568	NC_011248	NC_014008	NC_015957	NC_017421	NC_018876	NC_021506
NC_002754	NC_008569	NC_011249	NC_014009	NC_015958	NC_017422	NC_018877	NC_021507
NC_002755	NC_008570	NC_011250	NC_014010	NC_015959	NC_017423	NC_018878	NC_021508
NC_002758	NC_008571	NC_011251	NC_014011	NC_015963	NC_017424	NC_018879	NC_021514
NC_002771	NC_008573	NC_011252	NC_014012	NC_015964	NC_017425	NC_018880	NC_021515
NC_002774	NC_008576	NC_011253	NC_014013	NC_015966	NC_017426	NC_018881	NC_021516
NC_002927	NC_008577	NC_011254	NC_014014	NC_015967	NC_017427	NC_018882	NC_021517
NC_002928	NC_008578	NC_011255	NC_014017	NC_015968	NC_017428	NC_018883	NC_021518
NC_002929	NC_008593	NC_011256	NC_014019	NC_015969	NC_017429	NC_018884	NC_021519
NC_002932	NC_008595	NC_011257	NC_014022	NC_015970	NC_017430	NC_018885	NC_021520
NC_002935	NC_008596	NC_011258	NC_014023	NC_015974	NC_017431	NC_018886	NC_021521
NC_002936	NC_008598	NC_011259	NC_014025	NC_015975	NC_017432	NC_018887	NC_021525
NC_002937	NC_008599	NC_011260	NC_014026	NC_015976	NC_017433	NC_018888	NC_021526
NC_002939	NC_008600	NC_011261	NC_014027	NC_015977	NC_017434	NC_018889	NC_021527
NC_002940	NC_008601	NC_011262	NC_014028	NC_015978	NC_017435	NC_018936	NC_021528
NC_002942	NC_008607	NC_011263	NC_014029	NC_015979	NC_017436	NC_018937	NC_021552
NC_002944	NC_008608	NC_011264	NC_014030	NC_015980	NC_017437	NC_018938	NC_021553
NC_002945	NC_008609	NC_011265	NC_014031	NC_016001	NC_017438	NC_018939	NC_021554

NC_002946	NC_008610	NC_011274	NC_014032	NC_016002	NC_017439	NC_019012	NC_021555
NC_002947	NC_008611	NC_011281	NC_014033	NC_016010	NC_017440	NC_019042	NC_021577
NC_002950	NC_008618	NC_011282	NC_014034	NC_016011	NC_017441	NC_019048	NC_021591
NC_002951	NC_008639	NC_011283	NC_014035	NC_016012	NC_017442	NC_019100	NC_021592
NC_002952	NC_008686	NC_011294	NC_014039	NC_016021	NC_017443	NC_019272	NC_021593
NC_002953	NC_008687	NC_011295	NC_014041	NC_016022	NC_017444	NC_019382	NC_021594
NC_002967	NC_008688	NC_011296	NC_014098	NC_016023	NC_017445	NC_019386	NC_021657
NC_002971	NC_008696	NC_011297	NC_014100	NC_016024	NC_017446	NC_019387	NC_021658
NC_002973	NC_008697	NC_011311	NC_014103	NC_016025	NC_017447	NC_019388	NC_021659
NC_002976	NC_008698	NC_011312	NC_014106	NC_016026	NC_017448	NC_019391	NC_021661
NC_002977	NC_008699	NC_011313	NC_014107	NC_016027	NC_017449	NC_019392	NC_021662
NC_002978	NC_008700	NC_011314	NC_014108	NC_016028	NC_017450	NC_019393	NC_021663
NC_003028	NC_008701	NC_011315	NC_014109	NC_016029	NC_017451	NC_019394	NC_021668
NC_003030	NC_008702	NC_011316	NC_014117	NC_016030	NC_017452	NC_019395	NC_021669
NC_003037	NC_008703	NC_011333	NC_014118	NC_016034	NC_017453	NC_019396	NC_021670
NC_003042	NC_008704	NC_011334	NC_014119	NC_016035	NC_017454	NC_019397	NC_021709
NC_003047	NC_008705	NC_011350	NC_014120	NC_016037	NC_017455	NC_019425	NC_021710
NC_003062	NC_008709	NC_011351	NC_014121	NC_016041	NC_017456	NC_019427	NC_021711
NC_003063	NC_008710	NC_011352	NC_014122	NC_016043	NC_017457	NC_019428	NC_021712
NC_003064	NC_008711	NC_011353	NC_014125	NC_016046	NC_017458	NC_019429	NC_021713
NC_003065	NC_008712	NC_011355	NC_014131	NC_016047	NC_017459	NC_019430	NC_021714
NC_003078	NC_008713	NC_011365	NC_014132	NC_016048	NC_017460	NC_019431	NC_021715
NC_003080	NC_008726	NC_011366	NC_014133	NC_016050	NC_017461	NC_019432	NC_021716
NC_003098	NC_008738	NC_011367	NC_014134	NC_016051	NC_017462	NC_019433	NC_021717
NC_003103	NC_008739	NC_011368	NC_014135	NC_016052	NC_017464	NC_019434	NC_021718
NC_003106	NC_008740	NC_011369	NC_014136	NC_016070	NC_017465	NC_019435	NC_021721
NC_003112	NC_008741	NC_011370	NC_014144	NC_016077	NC_017467	NC_019436	NC_021722
NC_003116	NC_008750	NC_011371	NC_014145	NC_016078	NC_017468	NC_019437	NC_021723
NC_003131	NC_008751	NC_011374	NC_014147	NC_016079	NC_017469	NC_019438	NC_021725
NC_003132	NC_008752	NC_011375	NC_014148	NC_016109	NC_017470	NC_019439	NC_021726
NC_003134	NC_008757	NC_011386	NC_014149	NC_016110	NC_017471	NC_019440	NC_021727
NC_003140	NC_008758	NC_011407	NC_014150	NC_016111	NC_017472	NC_019537	NC_021728
NC_003143	NC_008759	NC_011408	NC_014151	NC_016112	NC_017473	NC_019551	NC_021729
NC_003155	NC_008760	NC_011411	NC_014152	NC_016113	NC_017474	NC_019552	NC_021730
NC_003197	NC_008761	NC_011413	NC_014153	NC_016114	NC_017475	NC_019556	NC_021731
NC_003198	NC_008762	NC_011415	NC_014154	NC_016115	NC_017476	NC_019560	NC_021732
NC_003210	NC_008763	NC_011416	NC_014155	NC_016146	NC_017477	NC_019561	NC_021733
NC_003212	NC_008764	NC_011419	NC_014157	NC_016147	NC_017479	NC_019562	NC_021734
NC_003228	NC_008765	NC_011420	NC_014158	NC_016148	NC_017480	NC_019563	NC_021738
NC_003240	NC_008766	NC_011498	NC_014159	NC_016149	NC_017481	NC_019564	NC_021739
NC_003241	NC_008767	NC_011499	NC_014160	NC_016150	NC_017482	NC_019565	NC_021740
NC_003267	NC_008769	NC_011526	NC_014165	NC_016445	NC_017483	NC_019566	NC_021741
NC_003270	NC_008770	NC_011527	NC_014166	NC_016446	NC_017484	NC_019567	NC_021742
NC_003272	NC_008771	NC_011528	NC_014168	NC_016510	NC_017485	NC_019670	NC_021743
NC_003273	NC_008781	NC_011529	NC_014169	NC_016511	NC_017486	NC_019673	NC_021744
NC_003276	NC_008782	NC_011561	NC_014170	NC_016512	NC_017487	NC_019674	NC_021745
NC_003277	NC_008783	NC_011562	NC_014171	NC_016513	NC_017488	NC_019675	NC_021807
NC_003295	NC_008784	NC_011563	NC_014172	NC_016514	NC_017490	NC_019676	NC_021810
NC_003296	NC_008785	NC_011564	NC_014205	NC_016515	NC_017491	NC_019677	NC_021811
NC_003317	NC_008786	NC_011565	NC_014206	NC_016516	NC_017492	NC_019678	NC_021812
NC_003318	NC_008787	NC_011566	NC_014207	NC_016582	NC_017493	NC_019679	NC_021813
NC_003361	NC_008789	NC_011567	NC_014209	NC_016584	NC_017495	NC_019680	NC_021814
NC_003364	NC_008790	NC_011585	NC_014210	NC_016585	NC_017496	NC_019681	NC_021815
NC_003366	NC_008791	NC_011586	NC_014211	NC_016586	NC_017497	NC_019682	NC_021816
NC_003383	NC_008800	NC_011593	NC_014212	NC_016587	NC_017499	NC_019683	NC_021817
NC_003384	NC_008816	NC_011595	NC_014213	NC_016588	NC_017501	NC_019684	NC_021818
NC_003385	NC_008817	NC_011601	NC_014214	NC_016589	NC_017502	NC_019685	NC_021819
NC_003413	NC_008818	NC_011602	NC_014215	NC_016590	NC_017503	NC_019686	NC_021820

NC_003425	NC_008819	NC_011603	NC_014216	NC_016591	NC_017504	NC_019689	NC_021821
NC_003450	NC_008820	NC_011653	NC_014217	NC_016592	NC_017505	NC_019690	NC_021822
NC_003454	NC_008825	NC_011654	NC_014218	NC_016593	NC_017506	NC_019691	NC_021823
NC_003485	NC_008826	NC_011655	NC_014219	NC_016594	NC_017507	NC_019692	NC_021824
NC_003551	NC_008835	NC_011656	NC_014220	NC_016595	NC_017508	NC_019693	NC_021825
NC_003552	NC_008836	NC_011657	NC_014221	NC_016596	NC_017509	NC_019694	NC_021826
NC_003869	NC_008942	NC_011658	NC_014222	NC_016597	NC_017510	NC_019695	NC_021827
NC_003888	NC_009004	NC_011660	NC_014225	NC_016598	NC_017511	NC_019696	NC_021828
NC_003901	NC_009007	NC_011661	NC_014226	NC_016599	NC_017512	NC_019697	NC_021829
NC_003902	NC_009008	NC_011662	NC_014228	NC_016600	NC_017513	NC_019698	NC_021830
NC_003903	NC_009009	NC_011663	NC_014230	NC_016601	NC_017514	NC_019699	NC_021831
NC_003904	NC_009012	NC_011664	NC_014246	NC_016602	NC_017515	NC_019700	NC_021832
NC_003909	NC_009033	NC_011665	NC_014248	NC_016603	NC_017516	NC_019701	NC_021833
NC_003910	NC_009035	NC_011666	NC_014249	NC_016604	NC_017517	NC_019702	NC_021834
NC_003911	NC_009036	NC_011667	NC_014250	NC_016605	NC_017518	NC_019703	NC_021837
NC_003912	NC_009037	NC_011668	NC_014251	NC_016606	NC_017519	NC_019727	NC_021838
NC_003919	NC_009038	NC_011720	NC_014253	NC_016607	NC_017520	NC_019728	NC_021839
NC_003921	NC_009040	NC_011721	NC_014254	NC_016608	NC_017521	NC_019729	NC_021840
NC_003922	NC_009049	NC_011722	NC_014256	NC_016609	NC_017522	NC_019730	NC_021841
NC_003923	NC_009050	NC_011723	NC_014257	NC_016610	NC_017523	NC_019731	NC_021842
NC_003980	NC_009051	NC_011724	NC_014258	NC_016612	NC_017524	NC_019732	NC_021843
NC_003981	NC_009052	NC_011725	NC_014259	NC_016613	NC_017525	NC_019733	NC_021844
NC_003997	NC_009053	NC_011726	NC_014297	NC_016614	NC_017526	NC_019734	NC_021845
NC_004041	NC_009073	NC_011727	NC_014298	NC_016616	NC_017527	NC_019735	NC_021846
NC_004061	NC_009074	NC_011728	NC_014299	NC_016617	NC_017528	NC_019736	NC_021847
NC_004070	NC_009075	NC_011729	NC_014300	NC_016618	NC_017529	NC_019737	NC_021848
NC_004088	NC_009076	NC_011730	NC_014301	NC_016619	NC_017530	NC_019738	NC_021869
NC_004113	NC_009077	NC_011731	NC_014302	NC_016620	NC_017531	NC_019739	NC_021870
NC_004116	NC_009078	NC_011732	NC_014303	NC_016621	NC_017532	NC_019740	NC_021871
NC_004129	NC_009079	NC_011733	NC_014304	NC_016622	NC_017533	NC_019741	NC_021872
NC_004193	NC_009080	NC_011734	NC_014305	NC_016623	NC_017534	NC_019742	NC_021877
NC_004252	NC_009083	NC_011735	NC_014306	NC_016624	NC_017535	NC_019743	NC_021878
NC_004253	NC_009084	NC_011736	NC_014307	NC_016625	NC_017536	NC_019744	NC_021879
NC_004307	NC_009085	NC_011737	NC_014308	NC_016626	NC_017537	NC_019745	NC_021880
NC_004310	NC_009089	NC_011738	NC_014309	NC_016627	NC_017538	NC_019746	NC_021881
NC_004311	NC_009091	NC_011739	NC_014310	NC_016628	NC_017539	NC_019747	NC_021882
NC_004319	NC_009092	NC_011740	NC_014311	NC_016629	NC_017540	NC_019748	NC_021883
NC_004320	NC_009135	NC_011741	NC_014313	NC_016630	NC_017541	NC_019749	NC_021884
NC_004337	NC_009136	NC_011742	NC_014314	NC_016631	NC_017542	NC_019750	NC_021885
NC_004342	NC_009138	NC_011743	NC_014315	NC_016632	NC_017543	NC_019751	NC_021886
NC_004343	NC_009140	NC_011744	NC_014316	NC_016633	NC_017544	NC_019752	NC_021887
NC_004344	NC_009142	NC_011745	NC_014317	NC_016634	NC_017545	NC_019753	NC_021888
NC_004347	NC_009226	NC_011747	NC_014318	NC_016635	NC_017546	NC_019754	NC_021889
NC_004349	NC_009227	NC_011748	NC_014319	NC_016636	NC_017547	NC_019755	NC_021890
NC_004350	NC_009228	NC_011749	NC_014323	NC_016637	NC_017548	NC_019756	NC_021891
NC_004368	NC_009229	NC_011750	NC_014328	NC_016638	NC_017549	NC_019757	NC_021892
NC_004369	NC_009230	NC_011751	NC_014329	NC_016639	NC_017550	NC_019758	NC_021893
NC_004431	NC_009253	NC_011753	NC_014330	NC_016640	NC_017551	NC_019759	NC_021894
NC_004432	NC_009254	NC_011757	NC_014331	NC_016641	NC_017552	NC_019760	NC_021895
NC_004459	NC_009255	NC_011758	NC_014332	NC_016642	NC_017553	NC_019761	NC_021896
NC_004460	NC_009256	NC_011760	NC_014333	NC_016645	NC_017554	NC_019762	NC_021897
NC_004461	NC_009257	NC_011761	NC_014334	NC_016646	NC_017555	NC_019763	NC_021898
NC_004463	NC_009328	NC_011766	NC_014335	NC_016745	NC_017556	NC_019764	NC_021899
NC_004545	NC_009329	NC_011768	NC_014355	NC_016746	NC_017557	NC_019765	NC_021900
NC_004547	NC_009332	NC_011769	NC_014363	NC_016747	NC_017558	NC_019766	NC_021902
NC_004551	NC_009337	NC_011770	NC_014364	NC_016748	NC_017559	NC_019770	NC_021903
NC_004552	NC_009338	NC_011771	NC_014365	NC_016749	NC_017560	NC_019771	NC_021904
NC_004554	NC_009339	NC_011772	NC_014366	NC_016750	NC_017561	NC_019772	NC_021905

NC_004555	NC_009340	NC_011773	NC_014370	NC_016751	NC_017562	NC_019773	NC_021906
NC_004556	NC_009341	NC_011774	NC_014371	NC_016768	NC_017563	NC_019774	NC_021907
NC_004557	NC_009342	NC_011775	NC_014374	NC_016771	NC_017564	NC_019775	NC_021908
NC_004565	NC_009343	NC_011776	NC_014375	NC_016772	NC_017565	NC_019776	NC_021909
NC_004567	NC_009344	NC_011777	NC_014376	NC_016773	NC_017566	NC_019777	NC_021910
NC_004572	NC_009345	NC_011778	NC_014377	NC_016774	NC_017567	NC_019778	NC_021911
NC_004578	NC_009346	NC_011779	NC_014378	NC_016775	NC_017568	NC_019779	NC_021912
NC_004603	NC_009347	NC_011780	NC_014387	NC_016776	NC_017569	NC_019780	NC_021913
NC_004604	NC_009348	NC_011781	NC_014388	NC_016777	NC_017570	NC_019789	NC_021915
NC_004605	NC_009349	NC_011782	NC_014389	NC_016778	NC_017571	NC_019790	NC_021916
NC_004606	NC_009350	NC_011783	NC_014390	NC_016779	NC_017572	NC_019791	NC_021917
NC_004631	NC_009376	NC_011784	NC_014391	NC_016780	NC_017573	NC_019792	NC_021918
NC_004632	NC_009377	NC_011785	NC_014392	NC_016781	NC_017574	NC_019793	NC_021919
NC_004633	NC_009378	NC_011830	NC_014393	NC_016782	NC_017575	NC_019814	NC_021920
NC_004663	NC_009379	NC_011831	NC_014394	NC_016783	NC_017576	NC_019815	NC_021921
NC_004668	NC_009380	NC_011832	NC_014408	NC_016784	NC_017577	NC_019842	NC_021976
NC_004669	NC_009381	NC_011833	NC_014409	NC_016785	NC_017578	NC_019845	NC_021977
NC_004670	NC_009425	NC_011834	NC_014410	NC_016786	NC_017579	NC_019846	NC_021978
NC_004671	NC_009426	NC_011835	NC_014414	NC_016787	NC_017580	NC_019847	NC_021979
NC_004703	NC_009427	NC_011836	NC_014448	NC_016788	NC_017581	NC_019848	NC_021984
NC_004704	NC_009428	NC_011837	NC_014471	NC_016789	NC_017582	NC_019849	NC_021985
NC_004719	NC_009429	NC_011852	NC_014472	NC_016790	NC_017583	NC_019892	NC_021986
NC_004720	NC_009430	NC_011878	NC_014479	NC_016791	NC_017584	NC_019893	NC_021987
NC_004721	NC_009431	NC_011879	NC_014483	NC_016792	NC_017585	NC_019894	NC_021988
NC_004722	NC_009432	NC_011880	NC_014484	NC_016793	NC_017586	NC_019895	NC_021989
NC_004741	NC_009433	NC_011881	NC_014494	NC_016794	NC_017587	NC_019896	NC_021990
NC_004757	NC_009434	NC_011882	NC_014495	NC_016795	NC_017588	NC_019897	NC_021991
NC_004829	NC_009436	NC_011883	NC_014497	NC_016796	NC_017589	NC_019898	NC_021992
NC_004838	NC_009437	NC_011884	NC_014498	NC_016797	NC_017590	NC_019902	NC_021993
NC_004842	NC_009438	NC_011885	NC_014499	NC_016798	NC_017591	NC_019903	NC_021994
NC_004851	NC_009439	NC_011886	NC_014500	NC_016799	NC_017592	NC_019904	NC_021995
NC_004917	NC_009440	NC_011887	NC_014501	NC_016800	NC_017593	NC_019905	NC_021996
NC_004923	NC_009441	NC_011888	NC_014502	NC_016801	NC_017594	NC_019906	NC_022000
NC_004924	NC_009442	NC_011889	NC_014503	NC_016802	NC_017595	NC_019907	NC_022001
NC_004925	NC_009443	NC_011890	NC_014504	NC_016803	NC_017596	NC_019908	NC_022040
NC_004943	NC_009445	NC_011891	NC_014506	NC_016804	NC_017617	NC_019936	NC_022041
NC_005003	NC_009446	NC_011892	NC_014507	NC_016805	NC_017618	NC_019937	NC_022042
NC_005004	NC_009453	NC_011893	NC_014532	NC_016806	NC_017619	NC_019938	NC_022043
NC_005005	NC_009454	NC_011894	NC_014533	NC_016807	NC_017620	NC_019939	NC_022044
NC_005006	NC_009455	NC_011895	NC_014534	NC_016808	NC_017621	NC_019940	NC_022045
NC_005007	NC_009456	NC_011896	NC_014535	NC_016809	NC_017622	NC_019941	NC_022046
NC_005008	NC_009457	NC_011898	NC_014537	NC_016810	NC_017623	NC_019942	NC_022047
NC_005027	NC_009464	NC_011899	NC_014538	NC_016811	NC_017624	NC_019943	NC_022048
NC_005042	NC_009465	NC_011900	NC_014539	NC_016812	NC_017625	NC_019949	NC_022049
NC_005043	NC_009466	NC_011901	NC_014540	NC_016813	NC_017626	NC_019950	NC_022050
NC_005061	NC_009467	NC_011916	NC_014541	NC_016814	NC_017627	NC_019951	NC_022051
NC_005070	NC_009468	NC_011958	NC_014543	NC_016815	NC_017628	NC_019952	NC_022075
NC_005071	NC_009469	NC_011959	NC_014544	NC_016816	NC_017629	NC_019953	NC_022078
NC_005072	NC_009470	NC_011960	NC_014548	NC_016817	NC_017630	NC_019954	NC_022079
NC_005085	NC_009471	NC_011961	NC_014549	NC_016818	NC_017631	NC_019955	NC_022080
NC_005090	NC_009472	NC_011962	NC_014550	NC_016819	NC_017632	NC_019956	NC_022081
NC_005125	NC_009473	NC_011963	NC_014551	NC_016820	NC_017633	NC_019957	NC_022082
NC_005126	NC_009474	NC_011969	NC_014552	NC_016821	NC_017634	NC_019958	NC_022083
NC_005128	NC_009475	NC_011971	NC_014554	NC_016822	NC_017635	NC_019959	NC_022084
NC_005139	NC_009477	NC_011973	NC_014555	NC_016823	NC_017636	NC_019960	NC_022092
NC_005140	NC_009478	NC_011978	NC_014556	NC_016824	NC_017637	NC_019961	NC_022093
NC_005213	NC_009479	NC_011979	NC_014558	NC_016825	NC_017638	NC_019962	NC_022097
NC_005229	NC_009480	NC_011981	NC_014560	NC_016826	NC_017639	NC_019963	NC_022106

NC_005230	NC_009481	NC_011982	NC_014561	NC_016827	NC_017640	NC_019964	NC_022107
NC_005231	NC_009482	NC_011983	NC_014562	NC_016828	NC_017641	NC_019965	NC_022108
NC_005232	NC_009483	NC_011984	NC_014563	NC_016829	NC_017642	NC_019966	NC_022109
NC_005241	NC_009484	NC_011985	NC_014614	NC_016830	NC_017643	NC_019967	NC_022110
NC_005244	NC_009485	NC_011986	NC_014616	NC_016831	NC_017644	NC_019968	NC_022111
NC_005295	NC_009486	NC_011987	NC_014618	NC_016832	NC_017645	NC_019969	NC_022112
NC_005296	NC_009487	NC_011988	NC_014621	NC_016833	NC_017646	NC_019970	NC_022113
NC_005297	NC_009488	NC_011989	NC_014622	NC_016834	NC_017647	NC_019971	NC_022114
NC_005303	NC_009494	NC_011990	NC_014623	NC_016835	NC_017648	NC_019972	NC_022115
NC_005362	NC_009495	NC_011991	NC_014624	NC_016836	NC_017649	NC_019973	NC_022116
NC_005363	NC_009496	NC_011992	NC_014625	NC_016837	NC_017650	NC_019974	NC_022117
NC_005364	NC_009497	NC_011993	NC_014626	NC_016838	NC_017651	NC_019975	NC_022118
NC_005707	NC_009504	NC_011994	NC_014628	NC_016839	NC_017652	NC_019976	NC_022119
NC_005773	NC_009505	NC_011995	NC_014629	NC_016840	NC_017653	NC_019977	NC_022120
NC_005791	NC_009506	NC_011996	NC_014632	NC_016841	NC_017654	NC_019978	NC_022121
NC_005810	NC_009507	NC_011997	NC_014633	NC_016842	NC_017655	NC_020050	NC_022123
NC_005813	NC_009508	NC_011998	NC_014634	NC_016843	NC_017656	NC_020051	NC_022124
NC_005814	NC_009511	NC_011999	NC_014638	NC_016844	NC_017657	NC_020052	NC_022125
NC_005815	NC_009512	NC_012000	NC_014639	NC_016845	NC_017658	NC_020053	NC_022126
NC_005816	NC_009513	NC_012001	NC_014640	NC_016846	NC_017659	NC_020054	NC_022130
NC_005823	NC_009515	NC_012002	NC_014641	NC_016847	NC_017660	NC_020055	NC_022132
NC_005824	NC_009516	NC_012003	NC_014642	NC_016848	NC_017661	NC_020056	NC_022196
NC_005835	NC_009517	NC_012004	NC_014643	NC_016854	NC_017662	NC_020057	NC_022198
NC_005838	NC_009523	NC_012026	NC_014644	NC_016855	NC_017663	NC_020059	NC_022221
NC_005861	NC_009524	NC_012028	NC_014650	NC_016856	NC_017664	NC_020060	NC_022222
NC_005863	NC_009525	NC_012029	NC_014651	NC_016857	NC_017665	NC_020061	NC_022223
NC_005871	NC_009565	NC_012030	NC_014652	NC_016858	NC_017668	NC_020062	NC_022224
NC_005877	NC_009566	NC_012032	NC_014654	NC_016859	NC_017669	NC_020063	NC_022225
NC_005916	NC_009567	NC_012034	NC_014655	NC_016860	NC_017670	NC_020064	NC_022226
NC_005945	NC_009613	NC_012036	NC_014656	NC_016861	NC_017671	NC_020075	NC_022227
NC_005951	NC_009614	NC_012037	NC_014657	NC_016862	NC_017672	NC_020076	NC_022228
NC_005955	NC_009615	NC_012039	NC_014658	NC_016863	NC_017673	NC_020089	NC_022234
NC_005956	NC_009616	NC_012040	NC_014659	NC_016864	NC_017675	NC_020124	NC_022236
NC_005957	NC_009617	NC_012108	NC_014664	NC_016884	NC_017717	NC_020125	NC_022237
NC_005966	NC_009619	NC_012109	NC_014666	NC_016885	NC_017718	NC_020126	NC_022238
NC_006055	NC_009620	NC_012115	NC_014718	NC_016886	NC_017719	NC_020127	NC_022239
NC_006085	NC_009621	NC_012121	NC_014719	NC_016887	NC_017720	NC_020128	NC_022241
NC_006086	NC_009622	NC_012124	NC_014720	NC_016888	NC_017721	NC_020129	NC_022244
NC_006087	NC_009632	NC_012125	NC_014721	NC_016893	NC_017722	NC_020130	NC_022245
NC_006128	NC_009633	NC_012128	NC_014722	NC_016894	NC_017723	NC_020133	NC_022246
NC_006129	NC_009634	NC_012129	NC_014723	NC_016900	NC_017724	NC_020134	NC_022247
NC_006138	NC_009635	NC_012130	NC_014724	NC_016901	NC_017725	NC_020135	NC_022248
NC_006139	NC_009636	NC_012131	NC_014727	NC_016902	NC_017726	NC_020156	NC_022268
NC_006140	NC_009637	NC_012133	NC_014728	NC_016903	NC_017727	NC_020157	NC_022269
NC_006142	NC_009641	NC_012166	NC_014729	NC_016904	NC_017728	NC_020163	NC_022270
NC_006153	NC_009648	NC_012169	NC_014731	NC_016905	NC_017729	NC_020164	NC_022271
NC_006154	NC_009649	NC_012177	NC_014732	NC_016906	NC_017730	NC_020165	NC_022347
NC_006155	NC_009650	NC_012180	NC_014733	NC_016907	NC_017731	NC_020180	NC_022348
NC_006156	NC_009651	NC_012185	NC_014734	NC_016908	NC_017732	NC_020181	NC_022349
NC_006177	NC_009652	NC_012204	NC_014735	NC_016909	NC_017733	NC_020182	NC_022350
NC_006270	NC_009653	NC_012207	NC_014736	NC_016911	NC_017734	NC_020195	NC_022351
NC_006274	NC_009654	NC_012214	NC_014737	NC_016912	NC_017735	NC_020196	NC_022352
NC_006297	NC_009655	NC_012225	NC_014738	NC_016913	NC_017736	NC_020207	NC_022353
NC_006298	NC_009656	NC_012226	NC_014749	NC_016914	NC_017737	NC_020208	NC_022354
NC_006300	NC_009659	NC_012416	NC_014750	NC_016915	NC_017738	NC_020209	NC_022355
NC_006322	NC_009660	NC_012417	NC_014751	NC_016928	NC_017739	NC_020210	NC_022356
NC_006347	NC_009661	NC_012438	NC_014752	NC_016929	NC_017740	NC_020211	NC_022357
NC_006348	NC_009662	NC_012439	NC_014753	NC_016930	NC_017741	NC_020212	NC_022358

NC_006349	NC_009663	NC_012440	NC_014754	NC_016931	NC_017742	NC_020229	NC_022359
NC_006350	NC_009664	NC_012441	NC_014755	NC_016932	NC_017743	NC_020230	NC_022360
NC_006351	NC_009665	NC_012442	NC_014756	NC_016933	NC_017761	NC_020238	NC_022361
NC_006360	NC_009667	NC_012466	NC_014758	NC_016934	NC_017762	NC_020239	NC_022362
NC_006361	NC_009668	NC_012467	NC_014759	NC_016935	NC_017763	NC_020240	NC_022364
NC_006362	NC_009669	NC_012468	NC_014760	NC_016936	NC_017764	NC_020241	NC_022369
NC_006363	NC_009670	NC_012469	NC_014761	NC_016937	NC_017765	NC_020242	NC_022370
NC_006365	NC_009671	NC_012470	NC_014762	NC_016938	NC_017766	NC_020243	NC_022371
NC_006366	NC_009672	NC_012471	NC_014763	NC_016939	NC_017768	NC_020244	NC_022436
NC_006368	NC_009673	NC_012472	NC_014774	NC_016940	NC_017769	NC_020245	NC_022437
NC_006369	NC_009674	NC_012473	NC_014796	NC_016941	NC_017770	NC_020246	NC_022438
NC_006370	NC_009675	NC_012483	NC_014797	NC_016942	NC_017771	NC_020247	NC_022439
NC_006371	NC_009697	NC_012488	NC_014800	NC_016943	NC_017772	NC_020248	NC_022440
NC_006373	NC_009698	NC_012489	NC_014801	NC_016944	NC_017773	NC_020249	NC_022441
NC_006375	NC_009699	NC_012490	NC_014802	NC_016945	NC_017774	NC_020250	NC_022442
NC_006376	NC_009700	NC_012491	NC_014803	NC_016946	NC_017775	NC_020260	NC_022443
NC_006377	NC_009704	NC_012520	NC_014804	NC_016947	NC_017776	NC_020261	NC_022444
NC_006389	NC_009705	NC_012521	NC_014810	NC_016948	NC_017777	NC_020262	NC_022513
NC_006390	NC_009706	NC_012522	NC_014811	NC_016972	NC_017778	NC_020263	NC_022514
NC_006391	NC_009707	NC_012523	NC_014812	NC_017017	NC_017779	NC_020264	NC_022515
NC_006392	NC_009708	NC_012526	NC_014814	NC_017018	NC_017780	NC_020265	NC_022516
NC_006393	NC_009712	NC_012527	NC_014815	NC_017019	NC_017781	NC_020266	NC_022521
NC_006394	NC_009713	NC_012528	NC_014816	NC_017020	NC_017782	NC_020267	NC_022523
NC_006395	NC_009714	NC_012529	NC_014817	NC_017021	NC_017783	NC_020268	NC_022524
NC_006396	NC_009715	NC_012552	NC_014818	NC_017022	NC_017784	NC_020269	NC_022525
NC_006397	NC_009717	NC_012559	NC_014819	NC_017023	NC_017785	NC_020272	NC_022526
NC_006448	NC_009718	NC_012560	NC_014820	NC_017024	NC_017786	NC_020273	NC_022528
NC_006449	NC_009719	NC_012563	NC_014824	NC_017025	NC_017787	NC_020274	NC_022529
NC_006461	NC_009720	NC_012577	NC_014825	NC_017026	NC_017788	NC_020275	NC_022530
NC_006462	NC_009725	NC_012578	NC_014826	NC_017027	NC_017789	NC_020276	NC_022531
NC_006463	NC_009726	NC_012579	NC_014827	NC_017028	NC_017790	NC_020283	NC_022532
NC_006509	NC_009727	NC_012580	NC_014828	NC_017029	NC_017791	NC_020284	NC_022533
NC_006510	NC_009749	NC_012581	NC_014829	NC_017030	NC_017792	NC_020285	NC_022534
NC_006511	NC_009767	NC_012582	NC_014830	NC_017031	NC_017793	NC_020286	NC_022535
NC_006512	NC_009776	NC_012583	NC_014831	NC_017032	NC_017794	NC_020287	NC_022536
NC_006513	NC_009777	NC_012586	NC_014833	NC_017033	NC_017795	NC_020288	NC_022537
NC_006526	NC_009778	NC_012587	NC_014834	NC_017034	NC_017796	NC_020289	NC_022538
NC_006529	NC_009779	NC_012588	NC_014836	NC_017035	NC_017797	NC_020290	NC_022539
NC_006530	NC_009780	NC_012589	NC_014837	NC_017038	NC_017798	NC_020291	NC_022540
NC_006569	NC_009782	NC_012590	NC_014838	NC_017039	NC_017799	NC_020292	NC_022541
NC_006570	NC_009783	NC_012622	NC_014839	NC_017040	NC_017800	NC_020293	NC_022542
NC_006576	NC_009784	NC_012623	NC_014840	NC_017041	NC_017801	NC_020294	NC_022543
NC_006578	NC_009785	NC_012624	NC_014841	NC_017042	NC_017802	NC_020296	NC_022544
NC_006582	NC_009786	NC_012625	NC_014842	NC_017043	NC_017803	NC_020297	NC_022545
NC_006624	NC_009787	NC_012626	NC_014844	NC_017044	NC_017804	NC_020298	NC_022546
NC_006625	NC_009788	NC_012631	NC_014908	NC_017045	NC_017805	NC_020299	NC_022547
NC_006629	NC_009789	NC_012632	NC_014909	NC_017046	NC_017806	NC_020300	NC_022548
NC_006663	NC_009790	NC_012633	NC_014910	NC_017047	NC_017807	NC_020301	NC_022549
NC_006672	NC_009791	NC_012634	NC_014911	NC_017048	NC_017808	NC_020302	NC_022550
NC_006673	NC_009792	NC_012654	NC_014914	NC_017049	NC_017809	NC_020303	NC_022551
NC_006674	NC_009793	NC_012655	NC_014915	NC_017050	NC_017810	NC_020304	NC_022566
NC_006675	NC_009794	NC_012656	NC_014916	NC_017051	NC_017811	NC_020305	NC_022567
NC_006676	NC_009795	NC_012657	NC_014917	NC_017052	NC_017812	NC_020306	NC_022568
NC_006677	NC_009796	NC_012658	NC_014918	NC_017053	NC_017813	NC_020307	NC_022569
NC_006814	NC_009800	NC_012659	NC_014920	NC_017054	NC_017814	NC_020308	NC_022570
NC_006823	NC_009801	NC_012660	NC_014921	NC_017055	NC_017815	NC_020376	NC_022571
NC_006824	NC_009802	NC_012667	NC_014922	NC_017056	NC_017816	NC_020377	NC_022575
NC_006831	NC_009806	NC_012668	NC_014923	NC_017057	NC_017817	NC_020378	NC_022576

NC_006832	NC_009828	NC_012669	NC_014924	NC_017058	NC_017818	NC_020379	NC_022578
NC_006833	NC_009829	NC_012673	NC_014925	NC_017059	NC_017819	NC_020380	NC_022579
NC_006834	NC_009831	NC_012686	NC_014926	NC_017060	NC_017820	NC_020381	NC_022582
NC_006840	NC_009832	NC_012687	NC_014931	NC_017061	NC_017821	NC_020382	NC_022583
NC_006841	NC_009837	NC_012691	NC_014932	NC_017062	NC_017822	NC_020383	NC_022584
NC_006842	NC_009838	NC_012695	NC_014933	NC_017063	NC_017831	NC_020384	NC_022587
NC_006855	NC_009839	NC_012704	NC_014934	NC_017064	NC_017832	NC_020385	NC_022588
NC_006856	NC_009840	NC_012718	NC_014935	NC_017065	NC_017834	NC_020386	NC_022591
NC_006873	NC_009848	NC_012720	NC_014957	NC_017066	NC_017845	NC_020387	NC_022592
NC_006905	NC_009850	NC_012721	NC_014958	NC_017067	NC_017846	NC_020388	NC_022593
NC_006908	NC_009879	NC_012723	NC_014960	NC_017068	NC_017847	NC_020389	NC_022594
NC_006932	NC_009881	NC_012724	NC_014961	NC_017069	NC_017848	NC_020390	NC_022600
NC_006933	NC_009882	NC_012725	NC_014962	NC_017070	NC_017856	NC_020391	NC_022601
NC_006958	NC_009883	NC_012726	NC_014963	NC_017071	NC_017857	NC_020392	NC_022602
NC_006969	NC_009897	NC_012730	NC_014964	NC_017072	NC_017858	NC_020393	NC_022603
NC_006970	NC_009900	NC_012731	NC_014965	NC_017073	NC_017860	NC_020394	NC_022604
NC_007005	NC_009901	NC_012732	NC_014966	NC_017074	NC_017861	NC_020409	NC_022605
NC_007086	NC_009921	NC_012751	NC_014970	NC_017075	NC_017866	NC_020410	NC_022606
NC_007103	NC_009922	NC_012752	NC_014972	NC_017076	NC_017867	NC_020411	NC_022607
NC_007104	NC_009925	NC_012759	NC_014973	NC_017077	NC_017903	NC_020417	NC_022608
NC_007105	NC_009926	NC_012778	NC_014974	NC_017078	NC_017904	NC_020418	NC_022610
NC_007106	NC_009927	NC_012779	NC_014975	NC_017079	NC_017905	NC_020419	NC_022648
NC_007107	NC_009928	NC_012780	NC_014976	NC_017080	NC_017906	NC_020420	NC_022649
NC_007109	NC_009929	NC_012781	NC_015052	NC_017081	NC_017907	NC_020421	NC_022650
NC_007110	NC_009930	NC_012782	NC_015053	NC_017082	NC_017909	NC_020422	NC_022651
NC_007111	NC_009931	NC_012785	NC_015057	NC_017092	NC_017910	NC_020449	NC_022653
NC_007146	NC_009932	NC_012790	NC_015058	NC_017093	NC_017911	NC_020450	NC_022654
NC_007164	NC_009933	NC_012791	NC_015059	NC_017094	NC_017912	NC_020453	NC_022655
NC_007168	NC_009934	NC_012792	NC_015060	NC_017095	NC_017919	NC_020503	NC_022656
NC_007169	NC_009937	NC_012793	NC_015061	NC_017096	NC_017920	NC_020504	NC_022657
NC_007170	NC_009939	NC_012794	NC_015062	NC_017098	NC_017921	NC_020505	NC_022658
NC_007171	NC_009943	NC_012795	NC_015063	NC_017100	NC_017922	NC_020506	NC_022659
NC_007181	NC_009952	NC_012796	NC_015064	NC_017101	NC_017923	NC_020507	NC_022660
NC_007204	NC_009953	NC_012797	NC_015065	NC_017102	NC_017924	NC_020508	NC_022661
NC_007205	NC_009954	NC_012803	NC_015066	NC_017103	NC_017925	NC_020509	NC_022662
NC_007274	NC_009955	NC_012804	NC_015067	NC_017104	NC_017926	NC_020510	NC_022663
NC_007275	NC_009956	NC_012806	NC_015125	NC_017105	NC_017927	NC_020511	NC_022664
NC_007292	NC_009957	NC_012807	NC_015136	NC_017106	NC_017933	NC_020512	NC_022665
NC_007294	NC_009958	NC_012808	NC_015137	NC_017107	NC_017934	NC_020513	NC_022737
NC_007295	NC_009959	NC_012809	NC_015138	NC_017108	NC_017935	NC_020514	NC_022738
NC_007296	NC_009972	NC_012810	NC_015144	NC_017109	NC_017941	NC_020515	NC_022739
NC_007297	NC_009973	NC_012811	NC_015145	NC_017110	NC_017942	NC_020516	NC_022759
NC_007298	NC_009974	NC_012814	NC_015146	NC_017111	NC_017943	NC_020517	NC_022760
NC_007322	NC_009975	NC_012815	NC_015147	NC_017112	NC_017944	NC_020518	NC_022777
NC_007323	NC_009976	NC_012846	NC_015151	NC_017113	NC_017945	NC_020519	NC_022778
NC_007332	NC_009997	NC_012847	NC_015152	NC_017114	NC_017946	NC_020520	NC_022780
NC_007333	NC_009998	NC_012848	NC_015153	NC_017115	NC_017949	NC_020521	NC_022781
NC_007335	NC_009999	NC_012849	NC_015155	NC_017116	NC_017950	NC_020522	NC_022782
NC_007336	NC_010000	NC_012850	NC_015160	NC_017117	NC_017951	NC_020523	NC_022783
NC_007337	NC_010001	NC_012851	NC_015161	NC_017118	NC_017952	NC_020524	NC_022784
NC_007347	NC_010002	NC_012852	NC_015162	NC_017119	NC_017953	NC_020525	NC_022785
NC_007348	NC_010003	NC_012853	NC_015163	NC_017120	NC_017954	NC_020526	NC_022786
NC_007349	NC_010008	NC_012854	NC_015164	NC_017121	NC_017955	NC_020527	NC_022792
NC_007350	NC_010009	NC_012855	NC_015165	NC_017122	NC_017956	NC_020528	NC_022793
NC_007351	NC_010010	NC_012856	NC_015166	NC_017123	NC_017957	NC_020529	NC_022794
NC_007352	NC_010063	NC_012857	NC_015167	NC_017124	NC_017958	NC_020530	NC_022795
NC_007354	NC_010067	NC_012858	NC_015168	NC_017125	NC_017959	NC_020531	NC_022806
NC_007355	NC_010070	NC_012880	NC_015169	NC_017126	NC_017960	NC_020532	NC_022807

NC_007356	NC_010079	NC_012881	NC_015170	NC_017127	NC_017961	NC_020533	NC_022808
NC_007384	NC_010080	NC_012883	NC_015172	NC_017128	NC_017962	NC_020534	NC_022873
NC_007385	NC_010084	NC_012891	NC_015174	NC_017129	NC_017963	NC_020535	NC_022874
NC_007404	NC_010085	NC_012892	NC_015177	NC_017130	NC_017964	NC_020536	NC_022875
NC_007406	NC_010086	NC_012912	NC_015178	NC_017131	NC_017965	NC_020537	NC_022876
NC_007410	NC_010087	NC_012913	NC_015179	NC_017132	NC_017966	NC_020538	NC_022877
NC_007411	NC_010102	NC_012914	NC_015180	NC_017133	NC_017986	NC_020539	NC_022878
NC_007412	NC_010103	NC_012917	NC_015181	NC_017134	NC_017999	NC_020540	NC_022879
NC_007413	NC_010104	NC_012918	NC_015182	NC_017135	NC_018000	NC_020541	NC_022880
NC_007414	NC_010115	NC_012923	NC_015183	NC_017136	NC_018001	NC_020542	NC_022881
NC_007426	NC_010117	NC_012924	NC_015184	NC_017137	NC_018002	NC_020543	NC_022882
NC_007427	NC_010118	NC_012925	NC_015185	NC_017138	NC_018010	NC_020544	NC_022883
NC_007428	NC_010120	NC_012926	NC_015186	NC_017139	NC_018011	NC_020545	NC_022884
NC_007429	NC_010123	NC_012943	NC_015187	NC_017140	NC_018012	NC_020546	NC_022886
NC_007430	NC_010124	NC_012947	NC_015188	NC_017141	NC_018013	NC_020547	NC_022898
NC_007432	NC_010125	NC_012960	NC_015189	NC_017142	NC_018014	NC_020548	NC_022899
NC_007434	NC_010157	NC_012961	NC_015213	NC_017143	NC_018015	NC_020549	NC_022900
NC_007435	NC_010158	NC_012962	NC_015214	NC_017144	NC_018016	NC_020551	NC_022901
NC_007481	NC_010159	NC_012967	NC_015215	NC_017145	NC_018017	NC_020553	NC_022902
NC_007482	NC_010160	NC_012968	NC_015216	NC_017146	NC_018018	NC_020555	NC_022903
NC_007483	NC_010161	NC_012969	NC_015217	NC_017147	NC_018019	NC_020556	NC_022904
NC_007484	NC_010162	NC_012970	NC_015218	NC_017148	NC_018020	NC_020557	NC_022905
NC_007486	NC_010163	NC_012971	NC_015219	NC_017149	NC_018021	NC_020558	NC_022906
NC_007487	NC_010167	NC_012972	NC_015221	NC_017150	NC_018022	NC_020559	NC_022907
NC_007488	NC_010168	NC_012973	NC_015222	NC_017151	NC_018023	NC_020560	NC_022908
NC_007489	NC_010169	NC_012982	NC_015223	NC_017152	NC_018024	NC_020561	NC_022909
NC_007490	NC_010170	NC_012983	NC_015224	NC_017153	NC_018025	NC_020562	NC_022910
NC_007491	NC_010172	NC_012984	NC_015258	NC_017154	NC_018026	NC_020563	NC_022911
NC_007492	NC_010175	NC_012985	NC_015259	NC_017155	NC_018027	NC_020564	NC_022912
NC_007493	NC_010180	NC_012987	NC_015275	NC_017156	NC_018028	NC_020565	NC_022913
NC_007494	NC_010181	NC_012988	NC_015276	NC_017157	NC_018065	NC_020566	NC_022964
NC_007498	NC_010182	NC_012989	NC_015277	NC_017158	NC_018066	NC_020567	NC_022991
NC_007503	NC_010183	NC_012997	NC_015278	NC_017159	NC_018067	NC_020568	NC_022997
NC_007504	NC_010184	NC_013008	NC_015291	NC_017160	NC_018068	NC_020796	NC_022998
NC_007505	NC_010263	NC_013009	NC_015311	NC_017161	NC_018069	NC_020797	NC_023001
NC_007506	NC_010278	NC_013010	NC_015312	NC_017162	NC_018073	NC_020798	NC_023002
NC_007507	NC_010280	NC_013016	NC_015313	NC_017163	NC_018077	NC_020799	NC_023003
NC_007508	NC_010287	NC_013037	NC_015314	NC_017164	NC_018078	NC_020800	NC_023004
NC_007509	NC_010296	NC_013061	NC_015315	NC_017165	NC_018079	NC_020801	NC_023010
NC_007510	NC_010320	NC_013062	NC_015318	NC_017166	NC_018080	NC_020802	NC_023011
NC_007511	NC_010321	NC_013093	NC_015319	NC_017167	NC_018081	NC_020812	NC_023012
NC_007512	NC_010322	NC_013118	NC_015320	NC_017168	NC_018089	NC_020813	NC_023013
NC_007513	NC_010331	NC_013119	NC_015321	NC_017169	NC_018092	NC_020814	NC_023018
NC_007514	NC_010333	NC_013123	NC_015322	NC_017170	NC_018101	NC_020815	NC_023019
NC_007515	NC_010334	NC_013124	NC_015376	NC_017171	NC_018106	NC_020816	NC_023024
NC_007516	NC_010335	NC_013129	NC_015377	NC_017172	NC_018107	NC_020817	NC_023025
NC_007517	NC_010336	NC_013130	NC_015378	NC_017173	NC_018108	NC_020819	NC_023028
NC_007519	NC_010337	NC_013131	NC_015379	NC_017174	NC_018139	NC_020820	NC_023029
NC_007520	NC_010338	NC_013132	NC_015380	NC_017175	NC_018140	NC_020821	NC_023030
NC_007530	NC_010364	NC_013156	NC_015381	NC_017176	NC_018141	NC_020822	NC_023031
NC_007575	NC_010366	NC_013157	NC_015382	NC_017177	NC_018142	NC_020823	NC_023032
NC_007576	NC_010367	NC_013158	NC_015383	NC_017178	NC_018143	NC_020824	NC_023033
NC_007577	NC_010368	NC_013159	NC_015385	NC_017179	NC_018145	NC_020825	NC_023035
NC_007595	NC_010369	NC_013160	NC_015386	NC_017180	NC_018146	NC_020826	NC_023036
NC_007604	NC_010371	NC_013161	NC_015387	NC_017181	NC_018147	NC_020827	NC_023037
NC_007606	NC_010373	NC_013162	NC_015388	NC_017182	NC_018148	NC_020828	NC_023044
NC_007607	NC_010374	NC_013163	NC_015389	NC_017183	NC_018149	NC_020829	NC_023045
NC_007608	NC_010376	NC_013164	NC_015390	NC_017184	NC_018150	NC_020830	NC_023057

NC_007613	NC_010379	NC_013165	NC_015391	NC_017185	NC_018177	NC_020831	NC_023060
NC_007614	NC_010380	NC_013166	NC_015407	NC_017186	NC_018178	NC_020832	NC_023061
NC_007615	NC_010381	NC_013167	NC_015408	NC_017187	NC_018179	NC_020833	NC_023062
NC_007616	NC_010382	NC_013168	NC_015409	NC_017188	NC_018180	NC_020834	NC_023063
NC_007617	NC_010394	NC_013169	NC_015410	NC_017189	NC_018181	NC_020887	NC_023064
NC_007618	NC_010395	NC_013170	NC_015416	NC_017190	NC_018182	NC_020888	NC_023065
NC_007622	NC_010396	NC_013171	NC_015417	NC_017191	NC_018183	NC_020891	NC_023066
NC_007624	NC_010397	NC_013172	NC_015418	NC_017192	NC_018184	NC_020892	NC_023069
NC_007626	NC_010398	NC_013173	NC_015419	NC_017193	NC_018185	NC_020894	NC_023073
NC_007633	NC_010399	NC_013174	NC_015420	NC_017194	NC_018186	NC_020895	NC_023075
NC_007641	NC_010400	NC_013190	NC_015421	NC_017195	NC_018187	NC_020907	NC_023076
NC_007643	NC_010401	NC_013191	NC_015422	NC_017196	NC_018188	NC_020908	NC_023134
NC_007644	NC_010402	NC_013192	NC_015423	NC_017199	NC_018189	NC_020909	NC_023135
NC_007645	NC_010403	NC_013193	NC_015424	NC_017200	NC_018190	NC_020910	NC_023136
NC_007650	NC_010404	NC_013194	NC_015425	NC_017201	NC_018191	NC_020911	NC_023137
NC_007651	NC_010407	NC_013198	NC_015426	NC_017202	NC_018192	NC_020912	NC_023138
NC_007677	NC_010408	NC_013199	NC_015427	NC_017203	NC_018193	NC_020913	NC_023139
NC_007678	NC_010410	NC_013200	NC_015428	NC_017204	NC_018194	NC_020929	NC_023140
NC_007681	NC_010418	NC_013201	NC_015429	NC_017205	NC_018195	NC_020930	NC_023141
NC_007705	NC_010424	NC_013202	NC_015430	NC_017206	NC_018196	NC_020931	NC_023142
NC_007712	NC_010465	NC_013203	NC_015431	NC_017207	NC_018197	NC_020932	NC_023143
NC_007713	NC_010466	NC_013204	NC_015433	NC_017208	NC_018219	NC_020933	NC_023144
NC_007714	NC_010467	NC_013205	NC_015434	NC_017209	NC_018220	NC_020934	NC_023145
NC_007715	NC_010468	NC_013206	NC_015435	NC_017210	NC_018221	NC_020935	NC_023146
NC_007716	NC_010469	NC_013207	NC_015436	NC_017211	NC_018222	NC_020936	NC_023147
NC_007717	NC_010470	NC_013208	NC_015437	NC_017212	NC_018223	NC_020937	NC_023148
NC_007718	NC_010471	NC_013209	NC_015458	NC_017214	NC_018224	NC_020938	NC_023149
NC_007719	NC_010473	NC_013210	NC_015459	NC_017215	NC_018225	NC_020939	NC_023150
NC_007720	NC_010474	NC_013211	NC_015460	NC_017216	NC_018227	NC_020940	NC_023151
NC_007722	NC_010475	NC_013212	NC_015461	NC_017217	NC_018265	NC_020941	NC_023497
NC_007759	NC_010476	NC_013213	NC_015470	NC_017218	NC_018266	NC_020942	NS_000191
NC_007760	NC_010477	NC_013214	NC_015471	NC_017219	NC_018267	NC_020943	NS_000192
NC_007761	NC_010478	NC_013215	NC_015474	NC_017220	NC_018268	NC_020944	NS_000193
NC_007762	NC_010479	NC_013216	NC_015475	NC_017221	NC_018285	NC_020945	NS_000194
NC_007763	NC_010480	NC_013222	NC_015496	NC_017222	NC_018286	NC_020946	NS_000195
NC_007764	NC_010482	NC_013223	NC_015497	NC_017223	NC_018287	NC_020947	NT_167350
NC_007765	NC_010483	NC_013224	NC_015498	NC_017224	NC_018288	NC_020948	NT_167351
NC_007766	NC_010485	NC_013235	NC_015499	NC_017225	NC_018289	NC_020949	NT_167352
NC_007775	NC_010486	NC_013260	NC_015500	NC_017226	NC_018290	NC_020950	NT_187069
NC_007776	NC_010487	NC_013263	NC_015501	NC_017227	NC_018291	NC_020951	NT_187070
NC_007777	NC_010488	NC_013264	NC_015508	NC_017228	NC_018293	NC_020952	NT_187071
NC_007778	NC_010498	NC_013265	NC_015510	NC_017229	NC_018294	NC_020953	NT_187072
NC_007779	NC_010501	NC_013282	NC_015511	NC_017230	NC_018299	NC_020954	NT_187073
NC_007790	NC_010502	NC_013283	NC_015512	NC_017231	NC_018303	NC_020955	NT_187074
NC_007791	NC_010503	NC_013284	NC_015513	NC_017232	NC_018304	NC_020956	NT_187075
NC_007792	NC_010504	NC_013285	NC_015514	NC_017233	NC_018405	NC_020957	NT_187076
NC_007793	NC_010505	NC_013315	NC_015516	NC_017234	NC_018406	NC_020958	NT_187077
NC_007794	NC_010506	NC_013316	NC_015517	NC_017235	NC_018407	NC_020959	NT_187078
NC_007795	NC_010507	NC_013353	NC_015518	NC_017236	NC_018408	NC_020960	NT_187079
NC_007796	NC_010508	NC_013354	NC_015519	NC_017237	NC_018409	NC_020961	NT_187080
NC_007797	NC_010509	NC_013355	NC_015520	NC_017238	NC_018410	NC_020962	NT_187081
NC_007798	NC_010510	NC_013356	NC_015554	NC_017239	NC_018411	NC_020963	NT_187082
NC_007799	NC_010511	NC_013357	NC_015555	NC_017240	NC_018412	NC_020964	NT_187083
NC_007801	NC_010512	NC_013358	NC_015556	NC_017241	NC_018413	NC_020965	NT_187084
NC_007802	NC_010513	NC_013361	NC_015558	NC_017242	NC_018414	NC_020966	NT_187085
NC_007880	NC_010514	NC_013362	NC_015559	NC_017243	NC_018415	NC_020967	NT_187086
NC_007899	NC_010515	NC_013363	NC_015560	NC_017244	NC_018416	NC_020968	NT_187087
NC_007900	NC_010516	NC_013364	NC_015561	NC_017245	NC_018417	NC_020969	NT_187088

NC_007901	NC_010517	NC_013365	NC_015562	NC_017246	NC_018418	NC_020970	NT_187089
NC_007907	NC_010518	NC_013366	NC_015563	NC_017247	NC_018419	NC_020971	NT_187090
NC_007908	NC_010519	NC_013367	NC_015564	NC_017248	NC_018420	NC_020972	NT_187091
NC_007912	NC_010520	NC_013368	NC_015565	NC_017249	NC_018421	NC_020973	NT_187092
NC_007925	NC_010524	NC_013369	NC_015566	NC_017250	NC_018422	NC_020974	NT_187093
NC_007929	NC_010525	NC_013370	NC_015567	NC_017251	NC_018423	NC_020975	NT_187094
NC_007930	NC_010528	NC_013385	NC_015571	NC_017252	NC_018485	NC_020976	NT_187095
NC_007940	NC_010529	NC_013386	NC_015572	NC_017253	NC_018486	NC_020977	NT_187096
NC_007941	NC_010530	NC_013406	NC_015573	NC_017254	NC_018487	NC_020978	NT_187097
NC_007946	NC_010531	NC_013407	NC_015574	NC_017255	NC_018488	NC_020979	NT_187098
NC_007947	NC_010539	NC_013408	NC_015576	NC_017256	NC_018489	NC_020980	NT_187099
NC_007948	NC_010541	NC_013409	NC_015577	NC_017257	NC_018490	NC_020981	NT_187100
NC_007949	NC_010542	NC_013410	NC_015578	NC_017258	NC_018491	NC_020982	NT_187101
NC_007950	NC_010543	NC_013411	NC_015579	NC_017259	NC_018492	NC_020983	NT_187102
NC_007951	NC_010544	NC_013412	NC_015580	NC_017260	NC_018493	NC_020984	NT_187103
NC_007952	NC_010545	NC_013416	NC_015581	NC_017261	NC_018494	NC_020985	NT_187104
NC_007953	NC_010546	NC_013418	NC_015582	NC_017262	NC_018495	NC_020986	NT_187105
NC_007954	NC_010547	NC_013419	NC_015583	NC_017263	NC_018496	NC_020987	NT_187106
NC_007955	NC_010549	NC_013421	NC_015588	NC_017264	NC_018497	NC_020988	NT_187107
NC_007958	NC_010550	NC_013422	NC_015589	NC_017265	NC_018498	NC_020989	NT_187108
NC_007959	NC_010551	NC_013438	NC_015590	NC_017266	NC_018499	NC_020990	NT_187109
NC_007960	NC_010552	NC_013440	NC_015591	NC_017267	NC_018500	NC_020992	NT_187110
NC_007961	NC_010553	NC_013441	NC_015592	NC_017268	NC_018501	NC_020993	NT_187111
NC_007963	NC_010554	NC_013442	NC_015593	NC_017269	NC_018502	NC_020995	NT_187112
NC_007964	NC_010555	NC_013446	NC_015594	NC_017270	NC_018503	NC_021002	NT_187113
NC_007968	NC_010556	NC_013450	NC_015595	NC_017271	NC_018507	NC_021003	NT_187114
NC_007969	NC_010557	NC_013451	NC_015596	NC_017272	NC_018508	NC_021004	NT_187115
NC_007971	NC_010571	NC_013452	NC_015597	NC_017273	NC_018509	NC_021005	NT_187116
NC_007972	NC_010572	NC_013453	NC_015598	NC_017274	NC_018510	NC_021006	NT_187117
NC_007973	NC_010577	NC_013454	NC_015600	NC_017275	NC_018511	NC_021007	NT_187118
NC_007974	NC_010578	NC_013456	NC_015601	NC_017276	NC_018512	NC_021008	NT_187119
NC_007984	NC_010579	NC_013457	NC_015602	NC_017277	NC_018513	NC_021009	NT_187120
NC_008009	NC_010580	NC_013501	NC_015603	NC_017278	NC_018514	NC_021010	NT_187121
NC_008010	NC_010581	NC_013502	NC_015632	NC_017279	NC_018515	NC_021011	NT_187122
NC_008011	NC_010582	NC_013504	NC_015633	NC_017280	NC_018516	NC_021012	NT_187123
NC_008012	NC_010602	NC_013505	NC_015634	NC_017281	NC_018517	NC_021013	NT_187124
NC_008013	NC_010604	NC_013508	NC_015635	NC_017282	NC_018518	NC_021014	NT_187125
NC_008014	NC_010605	NC_013509	NC_015636	NC_017283	NC_018520	NC_021015	NT_187126
NC_008021	NC_010606	NC_013510	NC_015637	NC_017284	NC_018521	NC_021016	NT_187127
NC_008022	NC_010609	NC_013511	NC_015638	NC_017285	NC_018522	NC_021017	NT_187128
NC_008023	NC_010610	NC_013512	NC_015656	NC_017286	NC_018524	NC_021018	NT_187129
NC_008024	NC_010611	NC_013515	NC_015657	NC_017287	NC_018525	NC_021019	NT_187130
NC_008025	NC_010612	NC_013516	NC_015658	NC_017288	NC_018526	NC_021020	NT_187131
NC_008027	NC_010617	NC_013517	NC_015659	NC_017289	NC_018527	NC_021021	NT_187132
NC_008036	NC_010622	NC_013518	NC_015660	NC_017290	NC_018528	NC_021022	NT_187133
NC_008042	NC_010623	NC_013519	NC_015661	NC_017291	NC_018529	NC_021023	NT_187134
NC_008043	NC_010625	NC_013520	NC_015662	NC_017292	NC_018531	NC_021024	NT_187135
NC_008044	NC_010627	NC_013521	NC_015663	NC_017293	NC_018532	NC_021025	NT_187136
NC_008048	NC_010628	NC_013522	NC_015664	NC_017294	NC_018533	NC_021026	NT_187137
NC_008054	NC_010629	NC_013523	NC_015665	NC_017295	NC_018580	NC_021028	NT_187138
NC_008060	NC_010630	NC_013524	NC_015666	NC_017296	NC_018581	NC_021030	NT_187139
NC_008061	NC_010631	NC_013525	NC_015667	NC_017297	NC_018582	NC_021031	NT_187140
NC_008062	NC_010632	NC_013526	NC_015670	NC_017298	NC_018583	NC_021033	NT_187141
NC_008086	NC_010633	NC_013530	NC_015671	NC_017299	NC_018584	NC_021035	NT_187142
NC_008087	NC_010634	NC_013531	NC_015672	NC_017300	NC_018585	NC_021036	NT_187143
NC_008095	NC_010635	NC_013532	NC_015673	NC_017301	NC_018586	NC_021038	NT_187144
NC_008118	NC_010644	NC_013552	NC_015674	NC_017303	NC_018587	NC_021039	NT_187145
NC_008119	NC_010645	NC_013592	NC_015675	NC_017304	NC_018588	NC_021040	NT_187146

NC_008120	NC_010655	NC_013595	NC_015676	NC_017305	NC_018589	NC_021041	NT_187147
NC_008121	NC_010656	NC_013596	NC_015677	NC_017306	NC_018590	NC_021042	NT_187148
NC_008122	NC_010657	NC_013597	NC_015678	NC_017307	NC_018591	NC_021043	NT_187149
NC_008146	NC_010658	NC_013642	NC_015679	NC_017308	NC_018592	NC_021044	NT_187150
NC_008147	NC_010659	NC_013654	NC_015680	NC_017309	NC_018593	NC_021046	NT_187151
NC_008148	NC_010660	NC_013655	NC_015681	NC_017310	NC_018594	NC_021047	NT_187152
NC_008149	NC_010672	NC_013656	NC_015682	NC_017311	NC_018604	NC_021049	NT_187153
NC_008150	NC_010673	NC_013657	NC_015683	NC_017312	NC_018605	NC_021050	NT_187154
NC_008209	NC_010674	NC_013665	NC_015684	NC_017313	NC_018606	NC_021051	NT_187155
NC_008212	NC_010676	NC_013714	NC_015685	NC_017314	NC_018607	NC_021052	NT_187156
NC_008213	NC_010677	NC_013715	NC_015686	NC_017315	NC_018608	NC_021054	NT_187157
NC_008226	NC_010678	NC_013716	NC_015687	NC_017316	NC_018609	NC_021055	NT_187158
NC_008228	NC_010679	NC_013717	NC_015688	NC_017317	NC_018610	NC_021056	NT_187159
NC_008229	NC_010680	NC_013718	NC_015689	NC_017318	NC_018611	NC_021057	NT_187160
NC_008230	NC_010681	NC_013719	NC_015690	NC_017319	NC_018612	NC_021058	NT_187161
NC_008242	NC_010682	NC_013720	NC_015693	NC_017320	NC_018618	NC_021059	NT_187162
NC_008243	NC_010683	NC_013721	NC_015694	NC_017321	NC_018619	NC_021060	NT_187163
NC_008244	NC_010688	NC_013722	NC_015695	NC_017322	NC_018620	NC_021064	NT_187164
NC_008245	NC_010693	NC_013729	NC_015696	NC_017323	NC_018621	NC_021066	NT_187165
NC_008253	NC_010694	NC_013730	NC_015697	NC_017324	NC_018622	NC_021081	NT_187166
NC_008254	NC_010695	NC_013731	NC_015698	NC_017325	NC_018623	NC_021082	NT_187167
NC_008255	NC_010696	NC_013732	NC_015699	NC_017326	NC_018624	NC_021083	NT_187168
NC_008258	NC_010697	NC_013733	NC_015700	NC_017327	NC_018625	NC_021084	NT_187169
NC_008260	NC_010698	NC_013734	NC_015701	NC_017328	NC_018626	NC_021085	
NC_008261	NC_010699	NC_013735	NC_015702	NC_017329	NC_018627	NC_021086	
NC_008262	NC_010715	NC_013736	NC_015703	NC_017330	NC_018630	NC_021089	
NC_008263	NC_010717	NC_013737	NC_015704	NC_017331	NC_018631	NC_021149	
NC_008264	NC_010718	NC_013738	NC_015705	NC_017332	NC_018632	NC_021150	

SUPPLEMENTARY FILE 2: PROOF-OF-CONCEPT PROTOCOLS

Case Study 1: Separating Viral Reads from Host Genomic Sequences.

Sample preparations and data generation: Isolation of Pseudomonas sp. phage ϕVader is fully described in Malki *et al*. (2015). Briefly, ϕVader was isolated from water samples collect from Chicago Lake Michigan nearshore waters. ϕVader propagates efficiently in culture with *Pseudomonas aeruginosa* ATCC 15692. 300μl of viral concentrate was treated with 5 μl of OPTIZYME^{TM} DNase I (Fisher BioReagents) for 30 minutes at 37°C in an effort to remove/reduce *P. aeruginosa* DNA. DNase was then inactivated via the addition of 50mM OPTIZYME EDTA at 65°C for 10 minutes. DNA was extracted using the MO BIO Laboratories UltraClean® Microbial DNA Isolation Kit and tested negative for *P. aeruginosa* contamination via targeted PCR of the 16S rRNA gene. Library construction (via the NEBNext Fragmentase and NEBNext® UltraTM DNA Library Prep kit) and sequencing (MiSeq Reagent Kit v2, 500 cycle) was conducted at the University of Texas Medical Branch (Galveston, TX).

Computational analysis: As neither the phage nor the laboratory host *Pseudomonas aeruginosa* ATCC 15692 have complete genomic sequences available. the RefSeq *P. aeruginosa* PAO1 genome [GenBank: NC_002516], excluding annotated bacteriophage coding regions, was used to separate host derived sequences from those from the phage genome. The genome sequence and annotation files were retrieved from NCBI. Coding regions within the bacterial chromosome or plasmid sequences which were annotated including the phrases “phage” (but not “macrophage”), “virus”, or “viral” were masked. Only paired-end reads in which neither read mapped to the background collection were retained for further processing.

Case Study 2: Isolating Viral Reads from Unknown Contaminating DNAs.

Sample preparations and data generation: The cyanophage ϕMHI42 was isolated as previously described (Watkins *et al*. 2014). Briefly, a surface water sample taken from a fishing lake was applied to exponential phage cultures of the cyanobacterium *Microcystis aeruginosa* BC 84/1. The presumptively infected culture was incubated for 24 hours, combined with molten agar and poured onto the surface of an agar plate containing a medium supportive to the general growth of cyanobacteria. All bacterial hosts used to propagate the phage were maintained in non-axenic culture, and contaminating DNA was present in large quantities from these associated bacteria, as opposed to the host itself. The formation of plaques indicated the presence of viruses, which were subsequently propagated and purified to obtain clonal phages. Clonal phage lysate was generated in liquid culture, and DNA extracted as previously described (Santos 1991). Extracted DNA was immobilized in high grade agarose and run on a PFGE gel as previously described (Wommack *et al*. 1999) in order to estimate genome size, which was approximately 150 kbp. Library construction and sequencing of concentrated DNA was performed by Macrogen Inc (Seoul, Rep. of Korea). The library was prepared using the Nextera® DNA Sample Preparation Kit and sequenced using the Illumina HiSeq platform, producing paired-end reads 100 nucleotides in length.

Computational analysis: Bacterial and plasmid sequences and annotations were retrieved from the NCBI FTP site's all.fna and all.ptt files (ftp://ftp.ncbi.nlm.nih.gov/genomes/Bacteria/), collected 09-Aug-14; those from the phylum *Cyanobacteria* were excluded to eliminate the possibility of false positives. Accession numbers for the sequences included are listed in Supplemental File 1. These sequences were then processed to mask annotated bacteriophage and viral coding regions as previously described. Raw ϕMHI42 were first assembled into contigs prior to analysis here. These contigs were then split into uniform subsequences of size 120bp (slightly larger than individual reads) with at minimum 60bp of overlap between subsequences. These "synthetic reads" were then mapped to their respective Bowtie2 index. It was necessary to process these reads differently due to the high level of bacterial contamination present in the WGS sequence data known to be present in ϕMHI42, and the likelihood that incorrect matches to cyanobacterial records would be made.

Case Study 3: Identifying Lysogenic Phages from Bacterial Populations.

Sample preparations and data generation: Water was collected from nearshore waters throughout the Chicago area during the summer 2014. No specific permits or permissions were required for the water samples collected from the Chicago Lake Michigan nearshore waters. 4L collections were filtered via successively smaller membranes, first a 0.45µm bottle-top cellulose acetate membrane filter (Corning Inc, Corning, NY) followed by a 0.22µm polyethersulfone membrane filter (MO BIO Laboratories, Carlsbad, CA). DNA was extracted using the MO BIO Laboratories PowerWater® DNA Isolation Kit (Carlsbad, CA). The protocol recommended by the manufacturer was followed with the exception of an additional heat treatment at 65°C for 10 minutes prior to initial vortexing. Library construction (via the Illumina Nextera® DNA Sample Prep Kit) and sequencing (MiSeq Reagent Kit v2, 300 cycle) was conducted at the Loyola University Chicago's Center for Biomedical Informatics, Maywood, IL).

Computational analysis: All bacterial and plasmid sequences and annotations were retrieved from the NCBI FTP site's all.fna and all.ptt files (ftp://ftp.ncbi.nlm.nih.gov/genomes/Bacteria/), collected 09-Aug-14. Accession numbers for the sequences included are listed in Supplemental File 1. Again, these sequences were then processed to mask annotated bacteriophage and viral coding regions. Sequence data was compared to this background and considered singleton reads.

References

Malki, K., Kula, A., Bruder, K., Sible, E., Hatzopoulos, T., Steidel, S., Watkins, S.C., & Putonti, C. (2015). Bacteriophages isolated from Lake Michigan demonstrate broad host-range across several bacteria phyla. *Virol. J.* **12,** 164**.**

Watkins, S., Smith, J., Hayes, P., & Watts, J. (2014). Characterisation of host growth after infection with a broad-range freshwater cyanopodophage. *PLoS One* **9,** e87339.

Santos, M.A. (1991). An improved method for the small scale preparation of bacteriophage DNA based on phage precipitation by zinc chloride. *Nuc Acids Res* **19,** 5442.

Wommack, K.E., Ravel, J., Hill, R.T., Chun, J., & Colwell, R.R. (1999). Population dynamics of Chesapeake Bay virioplankton: total-community analysis by Pulsed-Field Gel Electrophoresis. *Appl Environ Microbiol* **65,** 231-240.
